# A Study of the Association between SNP8NRG241930 in the 5' End of Neuroglin 1 Gene with Schizophrenia in a Group of Iranian Patients

**Published:** 2011-08-24

**Authors:** Seyed Ali Mohamad Shariati, Mehrdad Behmanesh, Hamid Galehdari

**Affiliations:** 1. Genetics Department, School of Biological Sciences, Tarbiat Modares University, Tehran, Iran; 2. Genetics Department, School of Basic Sciences, Chamran University, Ahwaz, Iran

**Keywords:** Schizophrenia, Association Study, Neuregulin1, Neuregulin1

## Abstract

**Objective:**

Neuregulin1 (*NRG1*) gene is among the most promising candidate genes for schizophrenia. This gene is located on 8p22-p12, a region with a reported linkage to schizophrenia. Several studies have reported an association between schizophrenia and the 5' end polymorphisms in this gene. However, some studies have failed to confirm the role of *NRG1* gene in the pathogenesis of schizophrenia. In the current study, we attempt to examine the association of SNP8NRG241930 from the *NRG1* gene with schizophrenia in an Iranian population. It is noteworthy that there has been no report on the *NRG1* association with schizophrenia in a population from the Middle East region.

**Materials and Methods:**

Genomic DNA samples were obtained via isolation from the peripheral blood cells of 95 unrelated subjects with schizophrenia and 95 matched healthy controls from southwest Iran. SNP8NRG241930 was genotyped by PCRRFLP using ScaI as a restriction endonuclease enzyme. Association of the SNP with schizophrenia was examined using the chi-square test. The frequency difference of alleles and genotypes between the two groups were compared. P≤0.05 was considered significant.

**Results:**

Statistical analysis on the studied polymorphism showed that both case and control groups were in Hardy-Weinberg equilibrium. The frequency of high risk allele (G allele) was 72.6% in patients, while this number was 56.8% in controls. The genotype frequencies in the patient group were as follows: GG (54%), GT (38%) and TT (8%) vs. genotype frequencies in the control group of: GG (26%), GT (63 %) and TT (11%).

**Conclusion:**

Considering allele and genotype frequencies, a significant association was observed between schizophrenia and SNP8NRG241930. The current study adds weight to the idea that some functional polymorphisms could exist in the 5' end of the NRG1 gene which increase susceptibility to schizophrenia. This is the first time that supportive evidence shows an involvement of the NRG1 locus in schizophrenia in an Iranian sample population.

## introduction

Schizophrenia is a severely disabling psychiatric disease that affects approximately 1% of the world’s population. It is one of the most heritable complex disorders, with persuasive evidence for genetic factors (1). Recent twin studies place the heritability of schizophrenia at more than 80% (2). Moreover, linkage analysis has identified several loci, including chromosome 8p, 13q, 22q and 6p that show linkage with schizophrenia. Several genes located on these regions have been studied extensively and there are supportive evidences for enrollment of these genes in the pathogenesis of schizophrenia (3).

Research into genetics of schizophrenia has found Neuregulin1 (*NRG1*) to be among the most promising candidate genes for this disease (4). *NRG1* is located on 8p, a region whose linkage with schizophrenia has been consistently reported (5). This gene spans 1.2 Mb and gives rise to many structurally and functionally distinct isoforms, through alternative promoter usage and alternative splicing. The isoforms are tissuespecifically expressed and differ significantly in their structure. The NRG1 proteins play crucial roles in the central nervous system and their functions in neurodevelopment and neuron plasticity, as well as pathological condition such as protection of the brain from damage induced by stroke and cancer has been confirmed in several studies (4, 6). Therefore, its position as well as function, strongly supports the *NRG1* gene as a susceptibility gene for neuropathological disorders such as bipolar disorder and schizophrenia (4, 7, 8). Several studies in different populations have positively reported the association of *NRG1* polymorphisms with schizophrenia. For the first time in 2002, Steafansson et al. suggested *NRG1* as a candidate susceptibility gene for schizophrenia in a linkage study carried out in an Icelandic population (9). They found a core at-risk haplotype, which is involved in the etiology of schizophrenia. This haplotype was composed of five SNP markers SNP8NRG241930, SNP8NRG243177, SNP8NRG433E1006, SNP8NRG221132 and SNP8NRG221533, and two microsatellite markers 478B14-848 and 420M9-1395 (9). Although the association of *NRG1* has been confirmed in several studies, there has been allelic heterogeneity between the different studies. Meanwhile, some studies have failed to replicate the association (10-13).

Genetic data from a new population is valuable because it addresses whether *NRG1* play a crucial role in predisposition to schizophrenia in a distinct population. In this report, we investigate the association of the SNP8NRG241930 polymorphism of *NRG1*, located at the 5' end of this gene, with schizophrenia in a homogenous population city of Ahwaz, southwestern, Iran. Recently, we have reported an association between schizophrenia and SNP8NRG221533 in the same population (14). Because of difficulties of sampling in this region and limited numbers of available patients, we could not increase our sample size to more than 95 patients in this study. PCR-RFLP was used for genotyping SNP8NRG241930. This polymorphism is a part of the Icelandic haplotype, originally found by Stefansson et al. in the Icelandic population (9). SNP8NRG241930 was selected for this study because it has shown significant association with schizophrenia as a single marker and because it was technically feasible to genotype this SNP by using restriction endonuclease enzymes (8, 15).

## Materials and Methods

### Case-control samples

Subjects were the patients of Salamat and Golestan Hospitals of Ahwaz, southwestern Iran. Subjects were interviewed by two independent experienced psychiatrists and each had a venous blood sample drawn for DNA extraction. Diagnoses were made according to the Diagnostic and Statistical Manual for Mental Disorder DSM-IV criteria (Table 1).

**Table 1:** The socio-demographic characteristics of cases and controls


	Controls (n=95)	Patients (n=95)
Continuous variable		
Age	38.34 ± 9.1	39.28 ± 8.29
Age of onset		22.02 ± 9.047
Discontinuous variable		
Female	30	31
Male	65	64
Educational level		
Illiteracy	2	14
Primary School	6	55
High School	72	21
College	15	5
Marital Status		
Single	24	70
Married	70	17
Divorced	1	8


For example, patients were entered into the study if they presented inclusion criteria such as delusion, hallucinations or disorganized speech symptoms for at least six months continuously. All patients had undergone repeated psychiatric hospitalizations. There was no case of familial relationship between patients and controls. Control subjects were drawn from the same population in southwestern Iran. Controls were accepted after they were interviewed by psychiatrists and had no history of hospitalization for psychiatric disorders, or no history of treatment for psychiatric illnesses themselves or in their family. Written informed consent was obtained before blood sampling. This study was approved by the Ethics Committee of Tarbiat Modares University and Jondi Shapour University of Medical Sciences.

### Genotyping

DNA was extracted from 100µl of whole blood using the DNPTM Kit (Cinnagen, Iran). Briefly, lysis solution was used to lyse blood cells. Subsequently DNA was selectively precipitated by isopropanol after which it was washed and desalted by ethanol. DNA was dissolved in double distilled water. The quantity and the quality of extracted DNA were examined spectrophotometrically or visually after electrophoresis in 1% agarose gel.

A pair of primers was designed using Primer3 software (Whitehead Institute, Cambridge, Massachusetts, USA). The primers were used for amplification of a 897 bp fragment which included SNP8NRG241930. The sequences of primers were as follows: forward, 5' AGAAGGGGAAGATTCAAGATGG 3' and reverse, 5' TCATGGACTGATGTGGCAATG 3'. Briefly, 100 ng of DNA were amplified for 35 cycles using recombinant *Taq* polymerase (Cinnagen, Iran). Following an initial 94℃ denaturing step for 3 minutes, samples were subjected to 35 cycles at 94℃ for 50 seconds, 56℃ for 45 seconds, 72℃ for 70 seconds and a final extension at 72℃ for 10 minutes.

**Fig 1:**
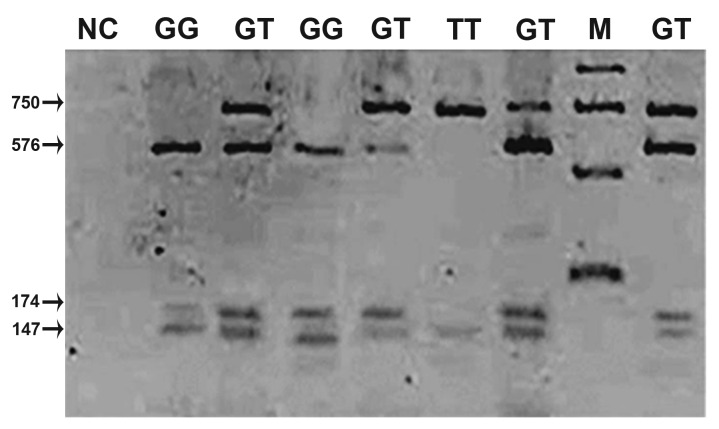
The result of used **PCR-RFLP** technique for genotyping of *SNP8NRG241930* . The full length of *PCR* fragment was 897bp. Digestion of the fragment with *ScaI* resulted in expected, 576bp, 174bp and 147bp fragment for **GG** genotype or 750bp and 147bp for TT genotype, respectively. The expected fragment for heterozygote individuals were 750 bp, 576bp , 174bp and 147 bp. *M*: means the *DNA* molecular weight size marker.

Afterwards, approximately 500 ng of amplified DNA was digested with Sca*I* (TAKARA, Japan) at 37℃ overnight. In case there was a G allele, the amplicon was cut into the 576 bp, 174 bp and 147 bp fragments. In case there was a T allele, we obtained the 750 bp and 147 bp fragments after digestion. The resulting products were analyzed by electrophoresis on a 1.2% agarose gel or 8% polyacrylamide gel (PAGE) following ethidium bromide staining (Fig 1).

**Fig 2:**
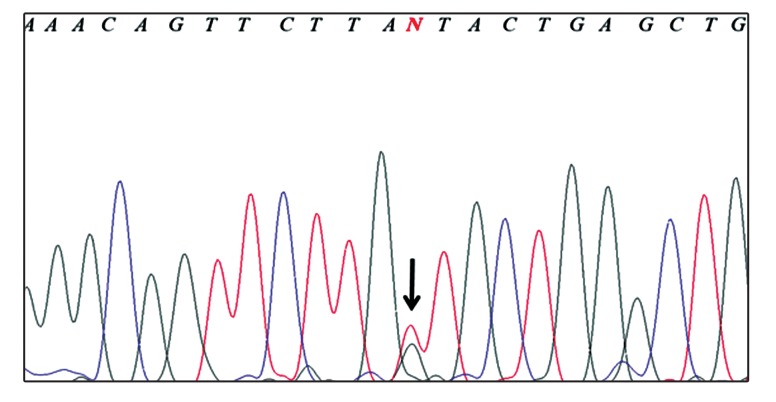
The results of **DNA** sequencing were consistent with determined genotype by **PCR-RLFP**. The picture shows the sequencing result for a heterozygote sample for **SNP8NRG241930**. The presence of two peaks at the **SNP** site indicates the heterozygosity of the sample

Finally, the accuracy of the method was confirmed by sequencing the randomly selected samples for each genotype (Fig 2).

### Statistical analysis

In this study, we tested the association of SNP8NRG241930 with schizophrenia as a single marker. The chi-square test was employed to compare frequency differences of the SNP8NRG241930 alleles and genotypes between the studied groups. A conventional p value of ≤0.05 was considered significant.

## Results

### Tissue MDA levels

We included 100 patients and 100 matched healthy individuals as controls in this study. From these, the genotypes of 95 cases matched with 95 control individuals for SNP8NRG241930 were determined. According to statistical analyses, the genotype frequencies were in Hardy-Weinberg equilibrium in both groups of controls (χ^2^=0.12, df = 2, p≤0.1) and patients (χ^2^=0.07, df =2, p≤ 0.1).

The frequency of the G allele in healthy individuals was 56.8% while this number for patients was 72.6%. The frequency of the G allele in the patients group was 1.27 fold higher compared with control subjects, which demonstrated a significant difference in allelic frequency.

**Fig 3:**
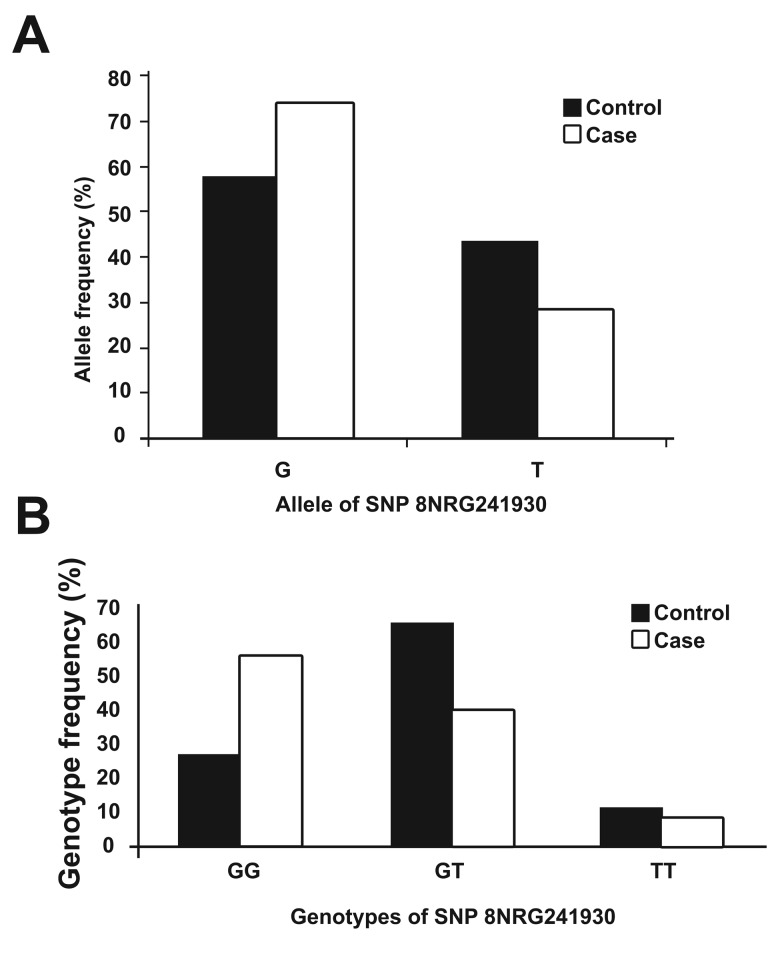
The allele and genotype ferquencies of SNP8NRG241930 in the studied groups. (A) Allelic frequencies and (B) genotype frequencies of single nucleotide polymorphisms between case and control groups.

Therefore, in confirmation with previous studies, a higher frequency of the G allele was observed in cases compared with the control group (Fig 3).

We compared the genotypic distribution of both groups and noted a significant difference in genotypic frequencies of patients compared with healthy individuals. In order to see if the presence of two risk alleles, in this case the G allele, could increase the risk of disease, we compared the frequency of homozygosity for the G allele with other genotypes in cases and controls (Table 2).

We found that homozygosity for the G allele was associated with increased risk of schizophrenia. Our analyses showed a significant difference between GG genotype versus GT and TT genotypes in the two groups. (χ^2^: 16.09, p≤0.001). This result indicated positive evidence for the association of *NRG1* with schizophrenia in our samples.

**Table 2:** Genotype distributions and allelic frequencies of SNP8NRG241930 among studied groups


GG vs. TT+GT			G vs. T alleles			TT	GT	GG	G allele frequency	Subjects (N)	SNPs ID
p	df	χ^2^	p	df	χ^2^	8%	38%	54%	72.6%	Cases (95)	SNP8NRG241930
≤0.001	1	16.09	≤0.001	1	10.375	11%	63%	26%	56.8%	Controls (95)	


df: Degree of freedom, χ^2^: chi square.

## Discussion

*NRG1* has been studied as a candidate gene for several diseases such as: schizophrenia, bipolar disorder (BPD), multiple sclerosis and cancer (6, 16-19). Compelling evidence has been gathered through association studies and functional analyses of the *NRG1* gene that indicate this gene as a strong susceptibility gene for schizophrenia (4).

Attempts to replicate association studies are of great value and have been proposed as a guideline to avoid spurious results. Importantly, replication of an association study in a new population can bring invaluable results about the role of a gene in the pathogenesis of a particular disease (20).

## Conclusion

Therefore, our results add weight to idea that there could be some functional variants in this region that play a crucial role in pathogenesis of schizophrenia. It is worth mentioning that the association of *NRG1* with schizophrenia in a demographically distinct population would be compelling evidence in favor of a true association between them.

Altogether, we believe that replication of association studies in a new population along with meta- analysis of data could be a major step towards understanding the role of genetic variations in the molecular pathology of schizophrenia. Our study suffers from small sample size and limited power. Therefore, studies with larger sample size are necessary to confirm the results of this study. This is a single marker association study but it would be interesting to include more SNPs and perform haplotype analysis of the 5' end of the *NRG1* gene in order to bring new evidence about the possible role of haplotypes of Hapice in the pathology of schizophrenia.
